# Incremental prognostic value of left atrial reservoir strain after ST-segment elevation myocardial infarction for the prediction of new-onset atrial fibrillation

**DOI:** 10.1007/s10554-025-03458-y

**Published:** 2025-07-05

**Authors:** Laima Caunite, Rinchyenkhand Myagmardorj, Xavier Galloo, Dorien Laenens, Jan Stassen, Takeru Nabeta, Idit Yedidya, Maria Chiara Meucci, Jurrien H. Kuneman, Inge J. van den Hoogen, Sophie E. van Rosendael, Hoi W. Wu, Victor M. van den Brand, Adrian Giuca, Karlis Trusinskis, Nina Ajmone Marsan, Jeroen J. Bax, Pieter van der Bijl

**Affiliations:** 1 Department of Cardiology, Leiden University Medical Center, Albinusdreef 2, Leiden, 2333 ZA The Netherlands; 2 Latvian Cardiology Center, Pauls Stradins Clinical University Hospital, Riga, Latvia; 3 Department of Cardiology, Vrije Universiteit Brussel (VUB), Universitair Ziekenhuis Brussel (UZ Brussel), Brussels, Belgium; 4 Department of Cardiology, Jessa Hospital, Hasselt, Belgium; 5 Department of Cardiology, Rabin Medical Center, Petah-Tikva, Israel; 6 Department of Cardiovascular Science, Fondazione Policlinico Universitario A. Gemelli IRCCS, Rome, Italy; 7 Department of Cardiology, Prof. Dr. C.C. Iliescu Emergency Institute for Cardiovascular Diseases, Bucharest, Romania; 8 Heart Centre, University of Turku and Turku University Hospital, Turku, Finland

**Keywords:** ST-segment elevation myocardial infarction, Left atrial function, Left atrial reservoir strain, Atrial fibrillation, Acute coronary syndrome

## Abstract

New-onset atrial fibrillation (AF) affects up to 21% of ST-segment elevation myocardial infarction (STEMI) patients. The value of LA reservoir strain to predict new-onset AF in a STEMI population has not been thoroughly investigated. We aimed to explore the incremental value of LA reservoir strain for predicting new-onset AF post-STEMI. Data were analyzed retrospectively from an ongoing STEMI registry. LA reservoir strain < 23% on transthoracic echocardiography was used as threshold for impaired LA function. The endpoint was new-onset AF. In total, 1238 patients (age 60 ± 12 years, 75% male) were included. After a median follow-up of 23 months, 92 (7.4%) patients developed new-onset AF. A similar prevalence of LA volume index ≥ 34 ml/m^2^ was seen between post-STEMI patients who developed new-onset AF and those who did not. In contrast, impaired LA reservoir strain was 1.5 times more common in individuals who developed AF (72% versus 48%; *p* < 0.001). Cumulative, event-free survival rates at five years in patients with preserved versus impaired LA reservoir strain were 93% versus 84%, respectively (log-rank χ2 = 19.81; *p* < 0.001). On multivariate Cox regression analysis LA reservoir strain remained significantly associated with new-onset AF (HR 0.97 (95% CI: 0.94–0.99); *p* = 0.025). Addition of LA reservoir strain provided incremental prognostic value over baseline clinical and echocardiographic risk markers (χ2 56.93 vs. 59.98; *p* = 0.013). Impaired LA reservoir strain was 1.5 times more common in patients who experienced new-onset AF post-STEMI, and was of incremental value for predicting the development of AF after adjusting for clinical and echocardiographic risk factors.

**Graphical abstract:**

Impaired LA reservoir strain was 1.5 times more common in patients who experienced new-onset AF post-STEMI, and was of incremental value for predicting the development of AF.

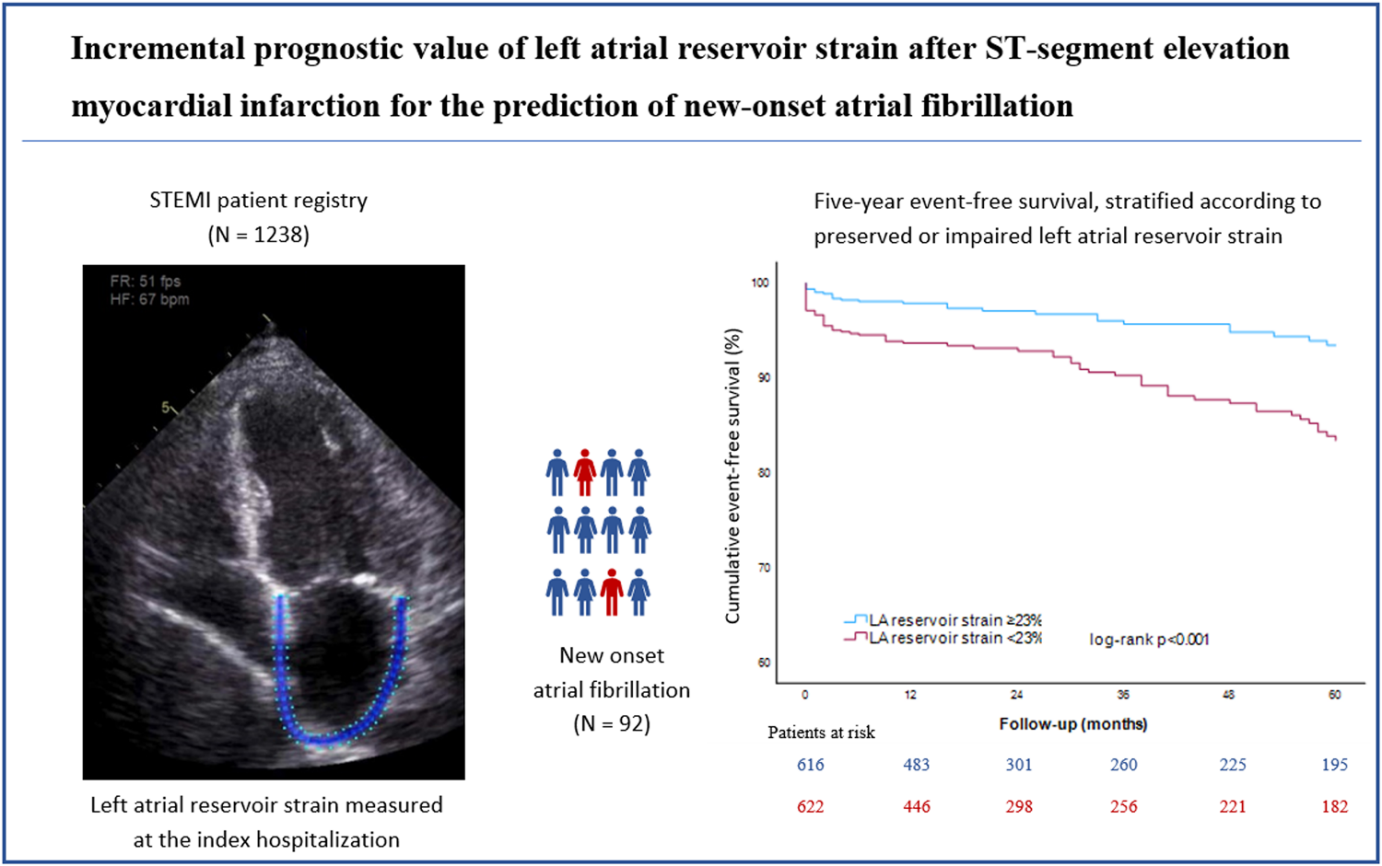

## Introduction

New-onset AF is common after ST-segment elevation myocardial infarction (STEMI), affecting up to 21% of all patients [1]. The combination of STEMI and AF is associated with high re-infarction and stroke rates [2] and worse quality of life [3, 4]. Detection of clinical, asymptomatic AF episodes can be challenging due to the intermittent sampling of 12-lead electrocardiograms and Holter studies. Longer term monitoring devices such as electrocardiogram patches and loop recorders remain expensive and not feasible to use for AF screening in all-comers [2]. An alternative strategy to identify STEMI patients who are at risk of AF development, and who may benefit from more frequent or in-depth screening, is needed.

The left atrium (LA) plays a key role in both the initiation and maintenance of AF [5]. Imaging of the LA is recognized as a key element in the characterization of the AF substrate [6]. Transthoracic echocardiography, which is widely available, safe and cost-effective, is indicated in all STEMI patients [1] and routinely includes measurements of LA size, e.g. left atrial volume index (LAVi). Not all patients who develop AF have an enlarged LA, and therefore assessment of LA function has the potential to provide additional insight into the substrate and consequences of AF. Results from the Effective aNticoaGulation with factor xA next GEneration in AF-Thrombolysis In Myocardial Infarction 48 (ENGAGE AF-TIMI 48) study demonstrated that LAVi remained normal in 36% of patients with known AF, but both LAVi and LA function were normal in only 17% of AF patients [7]. LA dysfunction has been previously demonstrated to precede LA enlargement in non-coronary artery disease patients [8]. The value of LA functional imaging in predicting new-onset AF has been demonstrated previously, e.g. in the Copenhagen City Heart study, where LA reservoir and conduit strain predicted incident AF in the general population [9]. Studies exploring LA strain in an acute MI population have, however suffered from a small size and few outcome events [10–12]. The aims of the current study were therefore to (1) analyze LA function in a large, contemporary STEMI database and (2) explore the incremental value of LA reservoir strain over conventional clinical and echocardiographic risk factors for the prediction of new-onset AF post-STEMI.

## Methods

### Patient population

The study included patients from an ongoing STEMI registry in the department of Cardiology, Leiden University Medical Center, The Netherlands [13]. All patients underwent primary percutaneous coronary intervention and were treated according to contemporary guidelines of the European Society of Cardiology [1]. Patients with a previous myocardial infarction (MI) and/or AF, a history of heart failure, severe valvular heart disease, suboptimal echocardiographic image quality disallowing LA reservoir strain analysis, AF during echocardiography or missing follow-up data, were excluded. Baseline clinical information, co-morbidities and coronary angiography findings were collected from the hospital information system (EPD-vision; Leiden University Medical Center, Leiden, The Netherlands). All patients underwent transthoracic echocardiography within 48 h of hospitalization for STEMI.

All data used in the current analysis were collected for routine clinical purposes and handled anonymously. The requirement for written informed consent was waived by the institutional review board on a patient level due to the retrospective design of the study.

### Transthoracic echocardiography

Transthoracic echocardiography images were acquired in the left lateral decubitus position using Vivid 7, E9 or E95 ultrasound systems (General Electric Vingmed Ultrasound, Horten, Norway). ECG-triggered images were stored in cine-loop format for offline analysis with EchoPac 202, 203 and 204 (General Electric Vingmed Ultrasound, Horten, Norway). Image analysis was performed by LC, RM, TN, IY, MCM and AG. According to current recommendations [14], left ventricular (LV) end-diastolic diameter and end-systolic diameter, interventricular septal thickness and posterior wall thickness were measured from the parasternal long-axis view. LV end-diastolic volume and LV end-systolic volume were measured and LV ejection fraction (LVEF) was calculated using the Simpson’s biplane method. Left ventricular mass was calculated by the cube formula from the two-dimensional long axis view [15] and indexed for body surface area. Pulsed-wave Doppler images were obtained in the apical four-chamber view at the tips of the mitral leaflets to measure the early (E) and late (A) peak diastolic velocities. E’ was calculated from the average septal and lateral e’ values acquired using pulsed-wave tissue Doppler imaging. LV global longitudinal strain (LVGLS) was calculated from apical two-chamber, three-chamber and four-chamber views by speckle-tracking analysis. LVGLS is expressed as absolute values. Right ventricular fractional area change and tricuspid annular plane systolic excursion were calculated from a right ventricle-focused apical four-chamber view. Pulmonary artery systolic pressure was estimated from the maximal tricuspid regurgitation velocity, combined with the diameter and respiratory collapse of the inferior vena cava.

### Left atrial measurements

LAVi was measured from the apical two- and four-chamber views using the Simpson’s biplane method and indexed to body surface area. LAVi ≥ 34 ml/cm^2^ was considered enlarged. LA reservoir strain was measured in the apical four-chamber view, where the LA endocardial border was traced from/to the mitral annulus, avoiding the pulmonary vein ostia and LA appendage [15]. Analysis was performed by dedicated speckle tracking software in images with frame rate of at least 40 frames per second. The start of the QRS complex was set as the reference point, and regions of interest were adjusted as required to ensure adequate tracking. If tracking of more than one segment was inadequate, the patient was excluded from further analysis.

### Clinical endpoint

Data on new-onset AF were collected by review of hospital records. New-onset AF was defined as the first documented episode either on 12-lead electrocardiogram or at least 30 s on Holter monitoring, or derived from loop recorder, implanted pacemaker, implantable cardioverter-defibrillator or cardiac resynchronization therapy devices. Follow-up was censored at the last outpatient visit to the Leiden University Medical Center, The Netherlands or at five years after the index hospitalization.

### Statistical analysis

Continuous variables are reported as mean ± standard deviation when normally distributed or as median and interquartile range when non-normally distributed. Categorical data are presented as frequencies and percentages. For between-group comparison the independent samples t-test was used if data were normally distributed, and the Mann-Whitney U test was used if data were non-normally distributed. Categorical variables were compared using the Pearson chi-square test. LA reservoir strain was dichotomized as normal/impaired by a previously established threshold of 23% [16, 17]. Cumulative, event-free survival was calculated by Kaplan-Meier analysis and compared between groups using a log-rank test. Univariate and multivariate Cox regression analyses were performed to assess the relationship between clinical and echocardiographic parameters in relation to the outcome. Due to a relatively small number of endpoint events, separate multivariable models were created for clinical and echocardiographic parameters with a P-value < 0.05 on univariate analysis. Thereafter significant parameters from both multivariable models were joined in a common model and lastly, LA reservoir strain was included. All echocardiographic parameters used in the Cox regression models were continuous variables. Collinearity was evaluated by the variance inflation factor, where values > 5 were interpreted as collinear [18]. In such a case, the parameter with a lower P-value on univariate analysis was chosen. To investigate the incremental value of LA reservoir strain over clinical and echocardiographic parameters to predict new-onset AF, a likelihood ratio test was performed. Statistical analysis was performed on SPSS version 25.0 (IBM, Armonk, New York, USA). All statistical tests were 2-sided, and a P-value < 0.05 was considered significant.

## Results

### Study population

From the initial 1389 patients, 121 were excluded because of a previous MI (*n* = 85), AF (*n* = 19), previously known heart failure (*n* = 17) and/or severe valvular pathology (*n* = 14). Furthermore, 13 patients had suboptimal image quality for LA reservoir strain analysis, four had AF at the time of transthoracic echocardiography and 13 were lost to follow-up, leaving 1238 patients as the final study population. Baseline demographic and clinical parameters are shown in Tables 1 and 2. The mean age was 60 ± 12 years and 930 (75%) were men. Patients who developed new-onset AF were older, had a higher prevalence of arterial hypertension, smoking and chronic obstructive pulmonary disease. Patients with impaired LA reservoir strain also were older, had a higher prevalence of diabetes mellitus, were less likely to be active smokers and had a higher body mass index, heart rate and peak troponin T values. Furthermore, these patients were more often prescribed diuretics upon discharge.

**Table 1:** Baseline clinical characteristics, stratified according to LA reservoir strain

Variable	Overall population (*N* = 1238)	Normal LA strain (*N* = 616)	Impaired LA strain (*N* = 622)	*P*-value
Age, years	60 ± 12	57 ± 11	62 ± 11	< 0.001
Men	930 (75.1%)	477 (77.4%)	453 (72.8%)	0.061
Arterial hypertension	462 (37.4%)	224 (36.4%)	238 (38.4%)	0.45
Dyslipidemia	243 (19.7%)	123 (20.0%)	120 (19.4%)	0.80
Family history of CAD	565 (46.2%)	303 (49.9%)	262 (42.5%)	0.009
Diabetes mellitus	111 (9%)	37 (6.0%)	74 (11.9%)	< 0.001
Current smoker	509 (41.1%)	275 (44.6%)	234 (37.6%)	0.012
COPD	43 (3.5%)	16 (2.6%)	27 (4.3%)	0.094
BMI, kg/m^2^	26.7 ± 4	26.5 ± 3.8	27.0 ± 4.2	0.038
Systolic BP, mmHg	134 ± 25	134 ± 25	134 ± 26	0.65
Heart rate, bpm	69 ± 12	67 ± 12	71 ± 13	< 0.001
QRS > 120 ms	71 (5.7%)	25 (4.1%)	46 (7.4%)	0.012
Peak troponin T, ng/l	3040 (1265; 6165)	2145 (890; 4488)	4220 (1865; 8075)	< 0.001
Peak CK, U/l	1191 (538; 2350)	948 (415; 1746)	1500 (722; 2930)	< 0.001
eGFR, ml/min/1.73m^2^	92 ± 24	94 ± 23	90 ± 26	0.004
LM/LAD as culprit	565 (45.6%)	265 (43.0%)	300 (48.2%)	0.066
Multivessel CAD	665 (53.7%)	324 (52.6%)	341 (54.8%)	0.43
Prescribed medication on discharge				
RAS-inhibitors	1155 (93.3%)	598 (97.1%)	601 (96.6%)	0.65
Beta-blockers	1180 (95.3%)	590 (95.8%)	590 (94.9%)	0.44
Diuretics	86 (6.9%)	19 (3.1%)	67 (10.8%)	< 0.001

Data are presented as mean ± standard deviation, median (interquartile range) and n (%)

Abbreviations: AF: atrial fibrillation; BP: blood pressure; BPM: beats per minute; BMI: body mass index; CAD: coronary artery disease; CK: creatine kinase; COPD: chronic obstructive pulmonary disease; eGFR: estimated glomerular filtration rate; LAD: left anterior descending coronary artery; LM: left main coronary artery; RAS: renin-angiotensin system

**Table 2:** Baseline clinical characteristics according to the presence of new-onset atrial fibrillation

Variable	Overall population (*N* = 1238)	New-onset AF (*N* = 92)	No AF (*N* = 1146)	*P*-value
Age, years	60 ± 12	67 ± 10	59 ± 11	< 0.001
Men	930 (75.1%)	74 (80.4%)	856 (74.7%)	0.22
Arterial hypertension	462 (37.4%)	43 (47.3%)	419 (36.6)	0.044
Dyslipidemia	243 (19.7%)	21 (22.8%)	222 (19.4%)	0.43
Family history of CAD	565 (46.2%)	41 (45.1%)	524 (46.2%)	0.83
Diabetes mellitus	111 (9%)	11 (12.0%)	100 (8.7%)	0.3
Current smoker	509 (41.1%)	25 (27.2%)	484 (42.2%)	0.005
COPD	43 (3.5%)	8 (8.7%)	35 (3.1%)	0.004
BMI, kg/m^2^	26.7 ± 4	26.7 ± 3.9	26.7 ± 4	0.89
Systolic BP, mmHg	134 ± 25	138 ± 28	134 ± 25	0.09
Heart rate, bpm	69 ± 12	72 ± 16	68 ± 12	0.04
QRS > 120 ms	71 (5.7%)	12 (13.0%)	59 (5.1%)	0.002
Peak troponin T, ng/l	3040 (1265; 6165)	4915 (2213; 8888)	2920 (1236; 5910)	< 0.001
Peak CK, U/l	1191 (538; 2350)	1738 (705; 3519)	1165 (535; 2253)	0.016
eGFR, ml/min/1.73m^2^	92 ± 24	83 ± 27	92 ± 24	< 0.001
LM/LAD as culprit	565 (45.6%)	56 (60.9%)	509 (44.4%)	0.002
Multivessel CAD	665 (53.7%)	56 (60.9%)	609 (53.1%)	0.15
Prescribed medication on discharge				
RAS-inhibitors	1155 (93.3%)	92 (95.7%)	1111 (96.9%)	0.49
Beta-blockers	1180 (95.3%)	86 (93.5%)	1094 (95.5%)	0.39
Diuretics	86 (6.9%)	16 (17.4%)	70 (6.1%)	< 0.001

Data are presented as mean ± standard deviation, median (interquartile range) and n (%)

Abbreviations:AF: atrial fibrillation; BP: blood pressure; BPM: beats per minute; BMI: body mass index; CAD: coronary artery disease; CK: creatine kinase; COPD: chronic obstructive pulmonary disease; eGFR: estimated glomerular filtration rate; LAD: left anterior descending coronary artery; LM: left main coronary artery; RAS: renin-angiotensin system

### Transthoracic echocardiography

Overall, the study population had preserved LVEF and normal right ventricular function (Tables 3 and 4). Patients who developed new-onset AF had worse LV systolic function, more impaired right ventricular systolic function, higher LV mass index and LV filling pressures. Even though mean LAVi was greater in the new-onset AF group, the proportion of patients with an enlarged LA was similar in both groups. LA reservoir strain was obtained from images with a mean frame rate of 60.8 ± 15.3 frames per second, measured on a random sample of *n* = 20 patients. The mean LA reservoir strain was 19.4 ± 8.4% in patients who developed new-onset AF and 24.2 ± 8.8% in patients who did not (*P* < 0.001). The new-onset AF group had a 1.5 times higher prevalence of impaired LA reservoir strain, according to the threshold of 23% (72.8% versus 48.4%; *P* < 0.001). Patients who had impaired LA reservoir strain also presented with larger LV dimensions and LV mass indices, worse LV and right ventricular systolic function, higher LV filling pressures and larger LAVI’s.

**Table 3:** Baseline echocardiographic characteristics, stratified according to LA reservoir strain

Variable	Overall population (*N* = 1238)	Normal LA strain (*N* = 616)	Impaired LA strain (*N* = 622)	*P*-value
LV EDD, mm	49 ± 5	49 ± 5	49 ± 5	0.013
LV ESD, mm	31 ± 6	31 ± 6	32 ± 6	< 0.001
IVST, mm	11.2 ± 1.4	11.0 ± 1.4	11.4 ± 1.4	< 0.001
PWT, mm	9.9 ± 1.2	9.9 ± 1.2	10.0 ± 1.2	0.04
LVMI, g/m^2^	97 ± 20	93 ± 19	100 ± 20	< 0.001
LV EDV, ml	141 ± 41	138 ± 40	144 ± 42	0.01
LV ESV, ml	10 ± 25	67 ± 22	75 ± 27	< 0.001
LVEF, %	50 ± 8	52 ± 7	49 ± 8	< 0.001
LVGLS, %	14 ± 3.4	15.1 ± 3.0	12.9 ± 3.5	< 0.001
LAVi, ml/m^2^	28.1 ± 8.9	27.1 ± 8.2	29.1 ± 9.4	< 0.001
LAVi ≥ 34 ml/m^2^	262 (21.2%)	103 (16.7%)	159 (25.6%)	< 0.001
E/A ratio	0.99 ± 0.38	1.00 ± 0.35	0.98 ± 0.42	0.46
E/E’ ratio	10.8 ± 3.9	10.1 ± 3.3	11.5 ± 4.3	< 0.001
RV FAC, %	43 ± 8.1	44 ± 8	42 ± 8	0.016
TAPSE, mm	20 ± 2.8	20 ± 2.8	20 ± 2.7	< 0.001
PASP, mmHg	25.8 ± 9.9	25 ± 9	26 ± 11	0.023
LA reservoir strain, %	23.8 ± 8.9	30.7 ± 6.8	17.0 ± 4.2	< 0.001

Data are presented as mean ± standard deviation and n (%)

Abbreviations: AF: atrial fibrillation; IVST: interventricular septal thickness; LA: left atrial; LAVi: left atrial volume index; LV EDD: left ventricular end-diastolic diameter; LV EDV: left ventricular end-diastolic volume; LVEF: left ventricular ejection fraction; LV ESD: left ventricular end-systolic diameter; LV ESV: left ventricular end-systolic volume; LVGLS: left ventricular global longitudinal strain; LVMI: left ventricular mass index; PASP: pulmonary artery systolic pressure; PWT: posterior wall thickness; RV FAC: right ventricular fractional area change; TAPSE: tricuspid annular plane systolic excursion

**Table 4:** Baseline echocardiographic characteristics according to the presence of new-onset atrial fibrillation

Variable	Overall population (*N* = 1238)	New-onset AF (*N* = 92)	No AF (*N* = 1146)	*P*-value
LV EDD, mm	49 ± 5	50 ± 5	49 ± 5	0.007
LV ESD, mm	31 ± 6	33 ± 6	31 ± 6	0.034
IVST, mm	11.2 ± 1.4	11.7 ± 1.5	11.2 ± 1.4	< 0.001
PWT, mm	9.9 ± 1.2	10.3 ± 1.3	9.9 ± 1.2	0.002
LVMI, g/m^2^	97 ± 20	107 ± 22	96 ± 20	< 0.001
LV EDV, ml	141 ± 41	147 ± 38	140 ± 41	0.16
LV ESV, ml	10 ± 25	75 ± 24	70 ± 25	0.056
LVEF, %	50 ± 8	49 ± 9	50 ± 8	0.057
LVGLS, %	14 ± 3.4	12.69 ± 3.6	14.1 ± 3.4	< 0.001
LAVi, ml/m^2^	28.1 ± 8.9	31.1 ± 11.7	27.9 ± 8.6	< 0.001
LAVi ≥ 34 ml/m^2^	262 (21.2%)	26 (28.3%)	236 (20.6%)	0.08
E/A ratio	0.99 ± 0.38	1.03 ± 0.49	0.98 ± 0.37	0.26
E/E’ ratio	10.8 ± 3.9	12.3 ± 3.8	10.7 ± 3.9	< 0.001
RV FAC, %	43 ± 8.1	41.3 ± 8.3	43.1 ± 8.1	0.037
TAPSE, mm	20 ± 2.8	19.3 ± 3.2	20.1 ± 2.8	0.012
PASP, mmHg	25.8 ± 9.9	29.6 ± 13.2	25.5 ± 9.5	< 0.001
LA reservoir strain, %	23.8 ± 8.9	19.4 ± 8.4	24.2 ± 8.8	< 0.001
LA reservoir strain < 23%	622 (50.2%)	67 (72.8%)	555 (48.4%)	< 0.001

Data are presented as mean ± standard deviation and n (%)

Abbreviations: AF: atrial fibrillation; IVST: interventricular septal thickness; LA: left atrial; LAVi: left atrial volume index; LV EDD: left ventricular end-diastolic diameter; LV EDV: left ventricular end-diastolic volume; LVEF: left ventricular ejection fraction; LV ESD: left ventricular end-systolic diameter; LV ESV: left ventricular end-systolic volume; LVGLS: left ventricular global longitudinal strain; LVMI: left ventricular mass index; PASP: pulmonary artery systolic pressure; PWT: posterior wall thickness; RV FAC: right ventricular fractional area change; TAPSE: tricuspid annular plane systolic excursion

### Outcomes

**Fig. 1:**
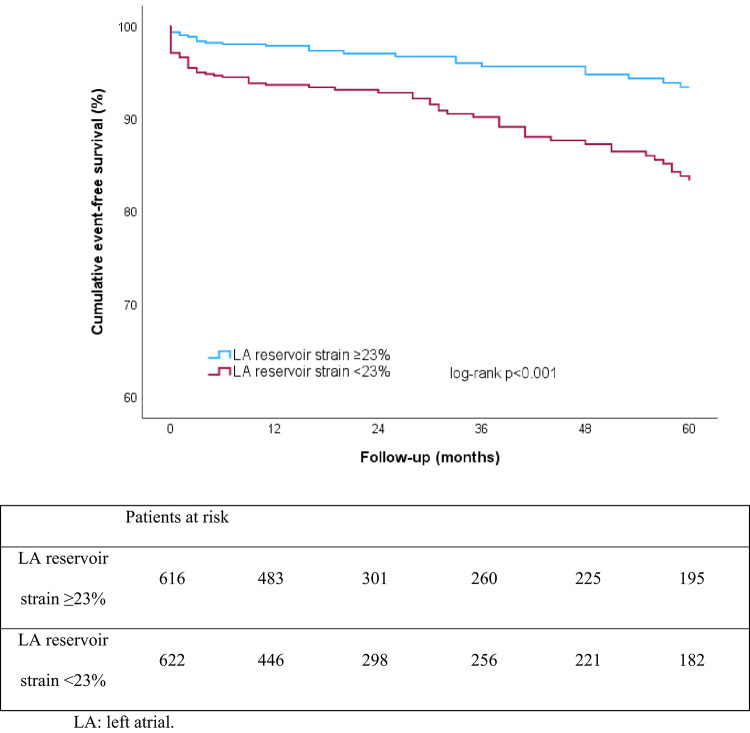
Kaplan-Meier curves for five-year event-free survival, stratified according to preserved or impaired left atrial reservoir strain

During a median follow-up of 23 months (IQR 11; 60) 92 (7.4%) patients developed new-onset AF; the majority (52; 56.5%) suffered the AF episode within the first year of follow-up. Cumulative, event-free survival rates at one, three and five years in patients with preserved versus impaired LA reservoir strain were 98%, 96% and 93% versus 94%, 90% and 84%, respectively (log-rank χ2 19.81; *P* < 0.001; Fig. 1).

On univariate Cox regression analysis age, smoking, chronic obstructive pulmonary disease, left main/left anterior descending coronary artery culprit lesions, a broad QRS (> 120 ms) complex, troponin T and creatine kinase levels and estimated glomerular filtration rate (eGFR) were associated with new-onset AF (Table 5). The results of the multivariate Cox regression analysis are shown in Table 6. A multivariate model with clinical variables (Model 1) was constructed from the aforementioned parameters, except for creatine kinase and smoking, which were excluded to avoid collinearity with troponin T and chronic obstructive pulmonary disease, respectively. Age and the left main or left anterior descending coronary artery as the culprit vessel remained independently associated with new-onset AF. The multivariate Cox regression model from echocardiographic parameters (Model 2) was constructed from LV end-diastolic diameter, interventricular septal thickness, LVGLS, LAVi, E/E’ ratio and pulmonary artery systolic pressure. Posterior wall thickness, LVEF and right ventricular fractional area change were not included in the model due to close correlation with interventricular septal thickness, LVGLS and right ventricular fractional area change, respectively. No significant collinearity was observed between LVGLS or LAVi and LA reservoir strain (variance inflation factor 1.2 and 1.01, respectively). In Model 2, LAVi, E/E’ ratio and pulmonary artery systolic pressure remained significantly associated with the outcome. Lastly, the combined model (Model 3) was constructed from parameters significant on clinical and echocardiographic models, and LA reservoir strain was added. In Model 3 age, left main or left anterior descending coronary artery as a culprit vessels, LAVi and LA strain remained significantly associated with new-onset AF.

**Table 5:** Univariate Cox regression analysis for new-onset atrial fibrillation

Variable	Univariate analysis	
	Hazard ratio (95% CI)	*P*-value
Age, per one year increase	1.06 (1.04–1.08)	< 0.001
Men	1.36 (0.81–2.28)	0.24
Arterial hypertension	1.49 (0.99–2.25)	0.059
Dyslipidemia	1.20 (0.74–1.95)	0.47
Diabetes mellitus	1.36 (0.73–2.56)	0.33
Current smoker	0.50 (0.32–0.79)	0.003
COPD	2.87 (1.39–5.94)	0.004
BMI, kg/m^2^	1.00 (0.94–1.05)	0.86
Systolic BP, per one mm Hg increase	1.01 (1.00–1.02)	0.09
QRS > 120 ms	2.31 (1.26–4.24)	0.007
Troponin T, per one ng/l increase	1.04 (1.02–1.07)	0.002
CK, per one U/l increase	1.13 (1.05–1.22)	0.002
eGFR, per one ml/min/1.73m^2^ increase	0.98 (0.97–0.99)	< 0.001
LAD/LM as culprit	1.88 (1.24–2.86)	0.003
Multivessel CAD	1.33 (0.87–2.02)	0.19
LV EDD per one mm increase	1.05 (1.01–1.09)	0.024
LV ESD, per one mm increase	1.03 (0.99–1.06)	0.12
IVST, per one mm increase	1.22 (1.07–1.39)	0.002
PWT, per one mm increase	1.27 (1.08–1.50)	0.004
LV EDV, per one ml increase	1.00 (1.00–1.01)	0.23
LV ESV, per one ml increase	1.01 (1.00–1.01)	0.14
LVEF, per 1% increase	0.98 (0.96–1.01)	0.18
LVGLS, per 1% increase	0.91 (0.86–0.96)	0.001
LAVi, per one ml/m^2^ increase	1.04 (1.02–1.06)	0.001
LAVi ≥ 34ml/m^2^	1.49 (0.95–2.35)	0.08
LVMI, per one g/m^2^ increase	1.02 (1.01–1.03)	< 0.001
E/A ratio per one unit increase	1.29 (0.80–2.10)	0.30
E/E’ ratio, per one unit increase	1.08 (1.03–1.13)	0.001
RV FAC, per 1% increase	0.98 (0.95–1.00)	0.041
TAPSE, per one mm increase	0.91 (0.85–0.99)	0.018
PASP, per one mm Hg increase	1.04 (1.02–1.06)	< 0.001
LA reservoir strain, per 1% increase	0.93 (0.91–0.96)	< 0.001

Abbreviations: BMI: body mass index; BP: blood pressure; CAD: coronary artery disease; CK: creatine kinase; COPD: chronic obstructive pulmonary disease; eGFR: estimated glomerular filtration rate; IVST: interventricular septal thickness; LA: left atrial; LAD: left anterior descending coronary artery; LAVi: left atrial volume index; LM: left main coronary artery; LV EDD: left ventricular end-diastolic diameter; LV EDV: left ventricular end-diastolic volume; LVEF: left ventricular ejection fraction; LV ESD: left ventricular end-systolic diameter; LV ESV: left ventricular end-systolic volume; LVGLS: left ventricular global longitudinal strain; LVMI: left ventricular mass index; PASP: pulmonary artery systolic pressure; PWT: posterior wall thickness; RV FAC: right ventricular fractional area change; TAPSE: tricuspid annular plane systolic excursion

**Table 6:** Multivariate Cox regression for new-onset atrial fibrillation

Variable	Model 1		Model 2		Model 3	
	Hazard ratio (95% CI)	*P*-value	Hazard ratio(95% CI)	*P*-value	Hazard ratio (95% CI)	*P*-value
Age, per one year increase	1.05 (1.03–1.08)	< 0.001			1.05 (1.02–1.07)	< 0.001
Current smoker	0.73 (0.46–1.18)	0.21				
COPD	2.02 (0.97–4.25)	0.06				
QRS > 120 ms	1.51 (0.81–2.81)	0.19				
Troponin T, per one ng/l increase	1.03 (1.00–1.06)	0.08				
eGFR, per one ml/min/1.73m^2^ increase	1.00 (0.99–1.01)	0.8				
LM/LAD as culprit	1.72 (1.11–2.66)	0.014			1,66 (1.05–2.61)	0.029
LV EDD, per one mm increase			1.04 (1.00–1.08)	0.059		
IVST, per one mm increase			1.15 (0.99–1.33)	0.063		
LVGLS, per 1% increase			0.99 (0.92–1.06)	0.78		
LAVi, per one ml/m^2^ increase			1.03 (1.01–1.05)	0.014	1.03 (1.01–1.05)	0.011
LVMI, per one g/m^2^ increase			1.00 (0.98–1.02)	>0.99		
E/E’ ratio, per one unit increase			1.06 (1.01–1.12)	0.027	1.01 (0.96–1.07)	0.65
TAPSE, per one mm increase			0.94 (0.87–1.03)	0.19		
PASP, per one mm Hg increase			1.03 (1.01–1.05)	0.008	1.02 (1.00–1.04)	0.07
LA reservoir strain, per 1% increase					0.97 (0.94–0.99)	0.025

Abbreviations: COPD: chronic obstructive pulmonary disease; eGFR: estimated glomerular filtration rate; IVST: interventricular septal thickness; LA: left atrium; LAD: left anterior descending coronary artery; LAVi: left atrial volume index; LM: left main coronary artery; LV EDD: left ventricular end-diastolic diameter; LVGLS: left ventricular global longitudinal strain; LVMI: left ventricular mass index; PASP: pulmonary artery systolic pressure; TAPSE: tricuspid annular plane systolic excursion

**Fig. 2:**
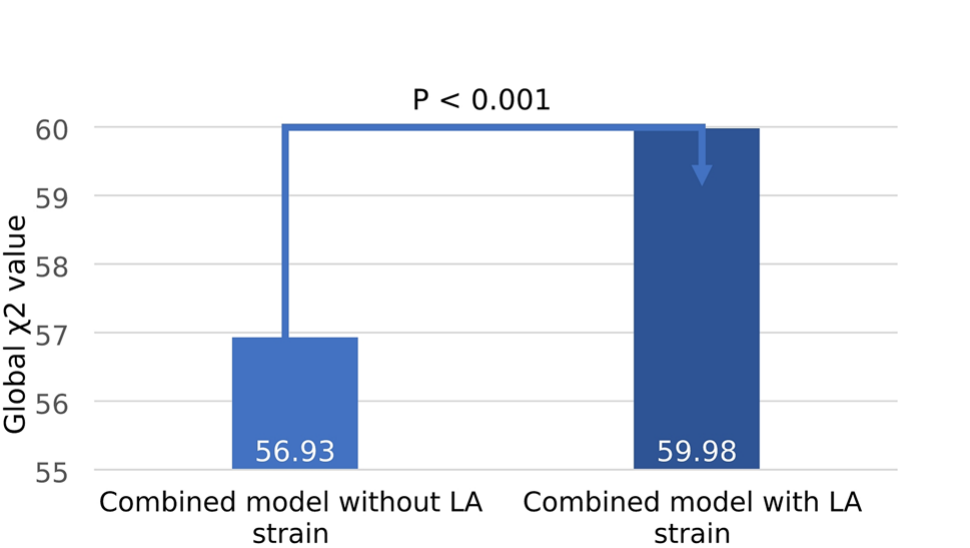
Incremental prognostic value of adding left atrial reservoir strain to a combined model of clinical risk factors, conventional echocardiographic parameters and left ventricular global longitudinal strain. Global χ2 values are shown for each model. Addition of left atrial reservoir strain resulted in significant improvement of the predictive value of the model. Combined model: Age, left main/left anterior descending coronary artery as culprit, left atrial volume index, E/E’ ratio, pulmonary artery systolic pressure. LA: left atrial

On likelihood ratio testing, the addition of LA reservoir strain provided incremental prognostic value over baseline clinical risk factors, conventional transthoracic echocardiography parameters and LVGLS (χ2 56.93 vs. 59.98; *P* = 0.013; Fig. 2).

## Discussion

The findings of the current study can be summarized as follows: (1) a similar prevalence of enlarged LAVi was seen between post-infarct patients who developed new-onset AF and those who did not, while in contrast, impaired LA reservoir strain was 1.5 times more common in patients who experienced new-onset AF, (2) the highest incidence of new-onset AF was observed within the first year after STEMI and (3) LA reservoir strain was independently associated with new-onset AF post-STEMI. Moreover, LA reservoir strain demonstrated incremental value over clinical and echocardiographic factors, including LAVi, for predicting new-onset AF post-infarct.

### Atrial fibrillation after STEMI

The estimated prevalence of AF in the general population ranges between 2 and 4% [2], while in STEMI patients it is reported to be as high as 21% [19]. In the current study, 7.4% of patients developed new-onset AF within five years after an MI, with more than half of the events occuring within the first year. Significantly higher rates of new onset AF have been observed in studies utilizing continuous rhythm monitoring devices [20–22]. The Implantable Cardiac Monitors in High-Risk Post-Infarction Patients with Cardiac Autonomic Dysfunction and Moderately Reduced Left Ventricular Ejection Fraction (SMART-MI-DZHK9) trial demostrated 23% prevalence of newly diagnosed AF during the median follow-up of 21 months [22]. Similarly, the Cardiac Arrhythmias and Risk Stratification After Acute Myocardial Infarction Study (CARISMA) found a 28% prevalence of at least six minute-long AF episodes [21]. A 58% prevalence of two minute-long AF episodes was observed in the Continuous Rhythm Monitoring in Patients After Acute Myocardial infaRction and pREServed Left venTricle Ejection Fraction (ARREST) study [20].

The high prevalence of AF in acute MI patients can be explained by the fact that coronary artery disease and AF share common risk factors, such as age, arterial hypertension, obesity and smoking [23, 24]. In addition, acute MI itself acts as a trigger for AF [25]. Regional and global wall motion abnormalities result in an immediate increase in LV pressure, volume and heart rate [25–27]. Myocardial edema may increase myocardial stiffness, also contributing to an increase in LV filling pressure [28]. This increases preload, which can result in LA dilatation [27, 29]. Furthermore, acute MI may directly contribute to new-onset AF through atrial ischemia, caused by coronary atrial branch occlusion [30], local electrolyte imbalances and alterations in the autonomic nervous system [25, 31]. These acute mechanisms also explain the high incidence of arrhythmias immediately following MI.

### Left atrial size and function in pathogenesis of atrial fibrillation

AF arises primarily from the LA. This chamber is predisposed to arrhythmia development by having thin walls, complex subendocardial fiber anatomy and direct exposure to LV pressures during diastole [29]. Increased preload causes LA cardiomyocyte replacement by fibrous tissue and electrical remodeling [5].

We found a similar prevalence of abnormal LAVi among patients with or without new-onset AF. Interestingly, some patients with new-onset AF had severe (> 48 ml/m^2^) LA enlargement. Since the probability of AF increases with LA volume [29], it raises the question whether these patients truly had new-onset AF, or if clinical, asymptomatic AF paroxysms had been present but were diagnosed through more frequent medical assessments after infarction. Another possibility is that the acute changes caused by STEMI, e.g. increased filling pressure, acted upon a preexisting substrate for AF, i.e. an enlarged LA. Notwithstanding the mechanism of LA enlargement, LAVi is not reliable enough for predicting which patients will develop AF post-infarct.

Previous studies have demonstrated the incremental value of measuring LA function for the identification of patients with acute MI at risk of AF [7, 12]. Although several LA function measures have been described, LA reservoir strain has gained traction due to the easy acquisition, sensitivity and excellent reproducibility [32]. The LA reservoir function phase starts at ventricular end-diastole and lasts until mitral valve opening [15], and has shown predictive value for new-onset AF in the general population [9], cryptogenic stroke [33] and heart failure with preserved EF [34]. Svartstein et al. [12] demonstrated a link between LA reservoir strain and new-onset AF in STEMI patients after adjusting for clinical risk factors and LVGLS or LAVi. Similarly, Beyls et al. [10] reported on the predictive value of LA reservoir strain after adjusting for LV function but not LA volume. The current study expands on these findings by demonstrating that LA reservoir strain is independently associated with new-onset AF in a large, homogenous STEMI patient population.

### Clinical implications

The current study demonstrates that impaired LA reservoir strain is associated with higher risk of new-onset AF after STEMI [2–4]. Undiagnosed AF carries a significant stroke risk. For example, Jaakola et al. reported that AF was first diagnosed during hospitalization in 20.8% of patients presenting with an ischemic stroke or transient ischemic attack [35]. The detection of clinical, asymptomatic paroxysmal AF can be challenging, but LA function measured by strain can serve as an indirect marker of patients who are at increased risk. LA strain measurement is simple and adds little time to an echocardiographic examination, which makes it a practical tool for identifying patients who would benefit most from more frequent or intensive screening for AF. Prospective studies which make use of continous rhythm monitoring, are required to determine the true value of LA strain in predicting post-infarct AF and its outcome implications.

### Limitations

This study is limited by its single-center, retrospective design and since continuous rhythm monitoring data were not systematically available for all patients, it is possible that some asymptomatic AF episodes were not registered. Nevertheless, the incidence of post-infarct AF is similar to previously reported studies [19]. A relatively low event total limited the number of covariates that could be included in the multivariate regression model, and we might not have been able to correct for all confounders. LA conduit and booster strains were not evaluated, although LA reservoir strain is the best validated deformation measure [15]. Thrombolysis in Myocardial Infarction flow, symptom-to-ballon and door-to-balloon times were not systematically available.

## Conclusions

A similar prevalence of enlarged LAVi was seen between post-infarct patients who developed new-onset AF and those who did not. Impaired LA reservoir strain was 1.5 times more common in patients who experienced new-onset AF an demonstrated incremental value for predicting the development of AF after adjusting for relevant clinical and echocardiographic risk factors. LA reservoir strain measurement is simple and adds little time to an echocardiographic examination, which makes it a practical tool for identifying patients who may benefit from surveillance for AF post-STEMI.

LA: left atrial.

## Data Availability

No datasets were generated or analysed during the current study.

## Abbreviations

AF: Atrial fibrillation
LA: Left atrium
LAVi: Left atrial volume index
LV: Left ventricle
LVEF: Left ventricular ejection fraction
LVGLS: Left ventricular global longitudinal strain
MI: Myocardial infarction
STEMI: ST-segment elevation myocardial infarction
